# A Deep Learning System to Predict the Histopathological Results From Urine Cytopathological Images

**DOI:** 10.3389/fonc.2022.901586

**Published:** 2022-05-24

**Authors:** Yixiao Liu, Shen Jin, Qi Shen, Lufan Chang, Shancheng Fang, Yu Fan, Hao Peng, Wei Yu

**Affiliations:** ^1^ Department of Urology, Peking University First Hospital, Peking University, Beijing, China; ^2^ Institute of Urology, Peking University, Beijing, China; ^3^ National Urological Cancer Center, Beijing, China; ^4^ School of Cyber Science and Technology, Beihang University, Beijing, China; ^5^ R&D Department, Yizhun Medical AI Co. Ltd, Beijing, China

**Keywords:** cyto-histo correlation, deep learning, urothelial carcinoma, urine cytology, convolutional neural network

## Abstract

**Background:**

Although deep learning systems (DLSs) have been developed to diagnose urine cytology, more evidence is required to prove if such systems can predict histopathology results as well.

**Methods:**

We retrospectively retrieved urine cytology slides and matched histological results. High-power field panel images were annotated by a certified urological pathologist. A deep learning system was designed with a ResNet101 Faster R-CNN (faster region-based convolutional neural network). It was firstly built to spot cancer cells. Then, it was directly used to predict the likelihood of the presence of tissue malignancy.

**Results:**

We retrieved 441 positive cases and 395 negative cases. The development involved 387 positive cases, accounting for 2,668 labeled cells, to train the DLS to spot cancer cells. The DLS was then used to predict corresponding histopathology results. In an internal test set of 85 cases, the area under the curve (AUC) was 0.90 (95%CI 0.84–0.96), and the kappa score was 0.68 (95%CI 0.52–0.84), indicating substantial agreement. The F1 score was 0.56, sensitivity was 71% (95%CI 52%–85%), and specificity was 94% (95%CI 84%–98%). In an extra test set of 333 cases, the DLS achieved 0.25 false-positive cells per image. The AUC was 0.93 (95%CI 0.90–0.95), and the kappa score was 0.58 (95%CI 0.46–0.70) indicating moderate agreement. The F1 score was 0.66, sensitivity was 67% (95%CI 54%–78%), and specificity was 92% (95%CI 88%–95%).

**Conclusions:**

The deep learning system could predict if there was malignancy using cytocentrifuged urine cytology images. The process was explainable since the prediction of malignancy was directly based on the abnormal cells selected by the model and can be verified by examining those candidate abnormal cells in each image. Thus, this DLS was not just a tool for pathologists in cytology diagnosis. It simultaneously provided novel histopathologic insights for urologists.

## Introduction

Urothelial carcinoma (UC) is one of the most common cancers worldwide (1). UCs are often multifocal and tend to recur. Thus, thorough screening and frequent surveillance are mandatory. The diagnosis of UC typically relies on the histopathological assessment of tissue resected by cystoscopy, yet it is an invasive approach and not easily accessible.

Urine cytology has played an important role in the screening and surveillance of UCs for many years for its effective, inexpensive, noninvasive nature (2–4). However, in the context of urine cytology, there is currently no gold standard for cyto-histo correlation in urine (5).

One possible solution could be to use the deep learning systems (DLSs) to build such links. DLSs have demonstrated a capacity superior to manual workflows in shifting through massive images to retrieve similar patterns and establish associations with novel traits in many medical data analysis tasks (6–8). Three studies have successfully automated the urine cytology diagnosis through the use of DLSs (9–11). One of these previous studies has further proven that DLSs might be able to determine the malignant potential of tumors more accurately than classical cytology (11). Researchers used a 16-layer convolutional neural network (CNN). Weights trained for initial UC cell detection were reused for the first 7 layers. New training with histopathological data started at the eighth layer. The DLS determined the presence of stromal invasion and performed a nuclear grading of tumor cells in the corresponding histological specimens. Therefore, DLSs have become a method that could potentially link cytopathology findings with histopathology results.

In this study, we hypothesized that routine urine cytology images contain information about the presence of malignant tissue in urinary tracts. The rationale for this cyto-histo correlation is that malignant tissues in urinary tracts undergo constant exfoliation, which sheds tumor cells and influences tumor cell morphology in urine. Building on those previous studies, we systematically investigated the presence of such correlation and aimed to capture it through our DLS. We trained and tested the DLS to spot UC cells in cytology images before using it to predict if a case would get malignant surgical pathology within the next 1 year. The results demonstrated that the DLS could predict the presence of malignancy and display such associations between cytopathology and histopathology through likelihood even without further training with histopathology data. Thus, DLS cytology can be used as not only a pathological tool to assist cytopathological diagnosis but also a novel risk-stratification tool to predict histopathology. This could help urologists make therapeutic decisions.

## Materials and Methods

### Data Acquisition

**Figure 1:**
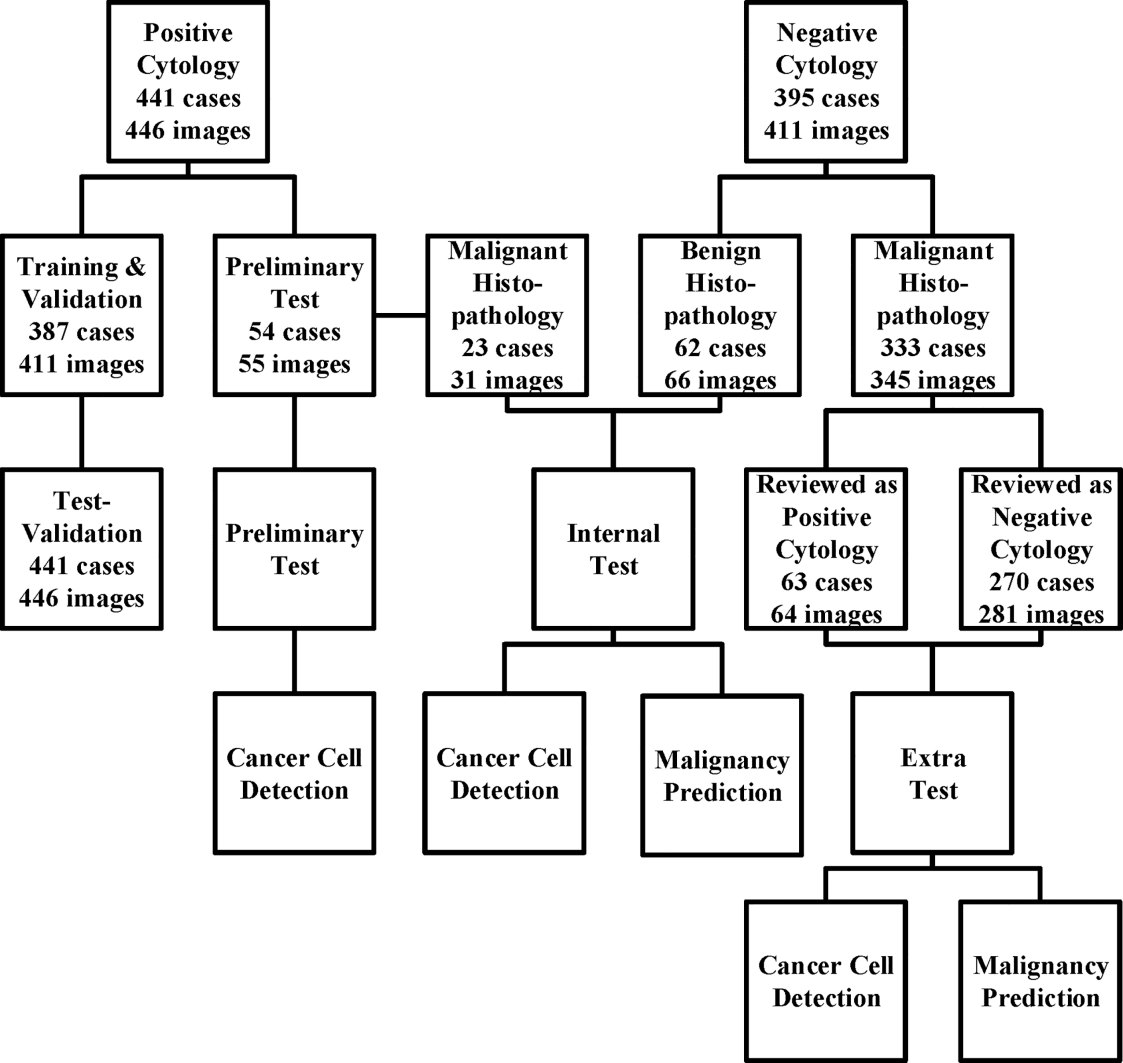
The data acquisition process is illustrated here. We collected the cytology images retrospectively and consecutively from Sep 2014 to Jan 2020 and built a series of data sets for training, validation, and tests. For those who underwent surgeries within the next 1 year, surgical results were also followed. The preliminary test set was only used for cancer cell detection, while the internal and extra test sets were used for both cancer cell detection and malignancy prediction.

All images were obtained from the archival glass of hematoxylin and eosin-stained urine cytocentrifugation cytology from consecutive patients who underwent examination, surgery, or both at Peking University First Hospital from 2014 to 2020 (**Figure 1**, **Table 1**). Urine cytology was routinely diagnosed using Papanicolaou’s classification at our institute (12). Classes III, IV, and V were defined as positive; class I and II (atypical) were defined as negative.

**Table 1:** Baseline characteristics.

	Training and Validation	Preliminary Test	Negative Cytology with Benign Histopathology	Negative Cytology with Malignant Histopathology
Age				
<60	79	10	26	26
≥60	308	44	36	307
Sex				
Female	260	11	23	95
Male	127	43	43	238
Cytology diagnosis^1^				
I	0	0	57	248
II	0	0	5	85
III	260	40	0	0
IV	127	14	0	0
V	0	0	0	0

^1^Cytology was diagnosed following Papanicolaou classification.

Among the 441 positive cases (patients diagnosed with UC based on cytologic examination), 211 received surgery (surgery within the next 1 year, if not otherwise clarified), all of which were diagnosed with UC based on histological examination (**Table 2**).

**Table 2:** Surgical follow-up.

	Training and Validation Set^1^	Positive Cytology with Malignant Histology	Negative Cytology with Benign Histology	Extra Test Set
N	188	23	62	333
Sex				
Female	63	5	19	95
Male	125	18	43	238
Age				
<60	26	3	26	74
≥60	162	20	36	259
Surgery^2^				
TUR-Bt or biopsy	102	16	48	310
nephroureterectomy	100	6	13	34
Radical cystectomy	9	1	1	20
Tumor				
Negative	0	0	62	0
upper urinary tract	89	6	0	15
Lower urinary tract	92	17	0	301
Synchronous U&L	7	0	0	17
tumor grade				
Low grade	24	7		117
High grade	164	16		213
NA^3^	0	0		3
Tumor stage				
Muscle non-invasive	104	16		277
Muscle invasive	82	6		52
NA^4^	2	1		4

Cytology was diagnosed following Papanicolaou criteria. Cancer grade was diagnosed using WHO2004. Tumor stage was diagnosed using TNM staging AJCC UICC 8th edition. Synchronous U&L, synchronous tumors in both upper and lower urinary tract. NA, not available.

^1^Only cases in the training and validation set who underwent surgery were listed here.

^2^Many cases undertook more than 1 procedure, either at one time or many times.

^3^Tumor grades were missing due to the following: a case reported as unable to rule out for low grade (n = 1); grade not reported for a case with in situ carcinoma (n = 1); a case reported as Grade 2 using WHO 1999 but not using WHO 2004 (n=1).

^4^Tumor stages were missing for those undertaken biopsies with no further operation available (n = 2 + 1 + 4).

Among the 395 negative cases (patients diagnosed with benign diseases based on cytologic examination), all received surgery, of which 333 were diagnosed with UC based on histological examination and the rest 62 were diagnosed with benign disease based on histological examination (**Table 2**). For the above 333 cases with contradicted cytopathological and histopathological results, a blinded pathologist’s review was carried out to check for overlooked cancer cells. As a result, 63 cases actually had cancer cells in their cytology images confirmed by a pathologist and should be deemed as positive cases.

From the original slides, 1,280 × 960-pixel, Joint Photographic Experts Group (jpeg) format images were exported: 466 images from positive cases and 417 images from negative cases. Subsequently, the training–validation set and preliminary test set, internal test set, and extra test set were defined by the different cytopathological and histopathological diagnoses. The training–validation set and preliminary test set were allocated by 8:1 stochastically. This study was approved by the institutional review board of Peking University First Hospital.

### Deep Learning System

**Figure 2:**
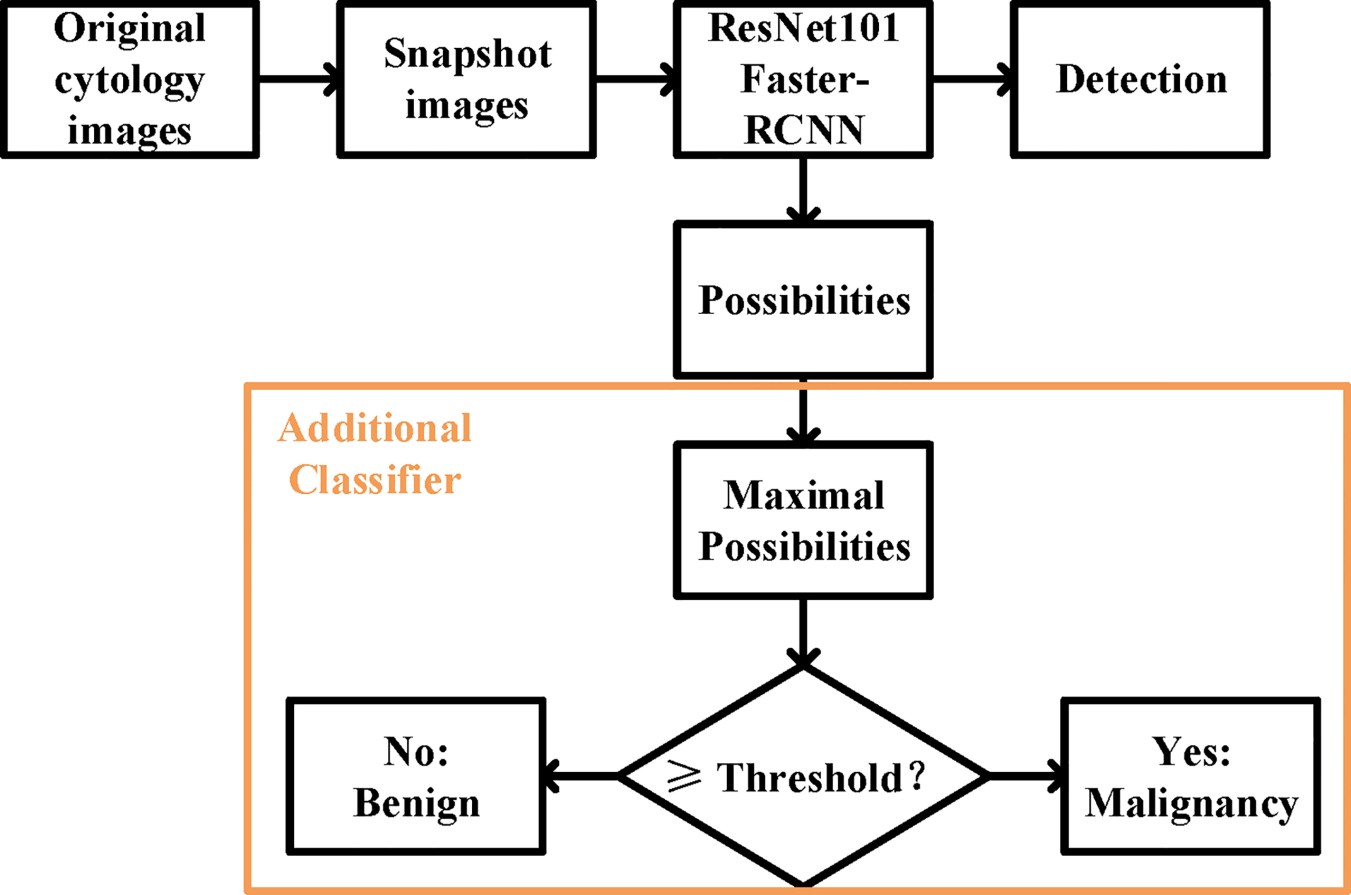
The overall design of the deep learning system. The ResNet 101 Faster-RCNN detected the UC cells while assigning a possibility to each of the cell, and an additional classifier picked the maximal possibility and predicted the histopathological malignant state according to the set threshold.

We built our DLS on a ResNet101 Faster-RCNN (**Figure 2**). ResNet101 is a 101-layer Reidual Network proposed on the 2016 IEEE Conference on Computer Vision and Pattern Recognition by He et al. (13). Deeper and restructured, ResNet101 has shown a high performance in many contexts of use including skin lesion detection and brain disease detection in magnetic resonance images (14, 15).

Faster-RCNN, short for faster region-based **c**onvolutional neural network, is a CNN that combines object detection and classification into one network (16). It extracts features, makes detections through these features, and quantifies the degree of fit at each detection using a value of possibility ranging from the worst of 0 to the optimum of 100. Evidence has shown that Faster-RCNN is especially good at detecting objects at multiple scales and aspect ratios, such as abnormal cervical cells in cytology images and cancer regions in colorectal biopsies (17, 18).

The model was implemented in Python 3.8 using TensorFlow (1.12.0) and Keras (2.0.3). Malignant cells with remarkable atypia in the jpeg images were annotated by a certified urological pathologist using the open-source software LabelMe (19). The images were then divided into 175 × 200-pixel panel subimages automatically, which were used for the training of the DLS.

ResNet101 was pretrained on the ImageNet database consisting of 1.2 million training images, with 1,000 classes of objects (20). The weight pretrained with ImageNet was used to initiate the weights of all convolutional layers, and all weights were trained with cytology images afterwards. The images passed through 33 convolution blocks and then through 1 dense layer. The SoftMax function was used as the activation function.

The training set and validation set were further allocated by 5:1 stochastically, for early stopping during network training to suppress overfitting. Spatial augmentation, including 90° rotation and vertical and horizontal flip, was applied in network training. We set 80 as the maximum epoch and stopped training if validation loss did not improve after 15 epochs.

For the prediction of malignancy, an additional classifier was added at the end of the initial DLS. The function of the classifier was to select the highest value of the possibilities in an image and to make a binary classification (benign or malignant) by comparing this value to the threshold (**Figure 2**). More details were provided in the **Supporting Materials (Supporting File 1)**.

### Evaluation Metrics

Performance was evaluated based on the testing results.

For the detection of UC cells, the annotations served as the reference standard. We used sensitivity, accuracy, and average false-positive cells per image. Sensitivities and accuracies were calculated using following formulas:


$$\text{Sensitivity}=\frac{\text{True Positives}}{\text{Total Annotations}}\;;\text{ Accuracy}=\frac{\text{True Positives}}{\text{True Positives }+\text{ False Positives}}$$

For the prediction of the malignancy, the surgical results served as the reference standard. We used sensitivity, specificity, the F1 score, and kappa score for evaluation. Cohen kappa scores reflect the agreement of the DLS with the pathologist reference standard (21). F1 scores were calculated using the following formula:


$$\text{F}1\text{ Score}=\frac{2\times \text{Precision}\times \text{Recall}}{\text{Precision}+\text{Recall}}$$

## Results

### Development of the Deep Learning System

The first step of our model is to detect just cancer cells. Therefore, we only annotated the cancer cells in the jpeg images for training and validation purposes, and treated all the other cells in the same image as background (**Supporting File1**). In total, 1,364 cells from 411 1,280 × 960-pixel images were labelled. A total of 1953 subimages were obtained. Each subimage contained at least one label in it. Sub-images were subsequently randomly allocated into the training and validation sets by the ratio of 5:1, and subjected to the pretrained DLS, as mentioned in *Materials and Methods*. Both the total loss and the system accuracy stabilized after 45–50 epochs for the validation set. A final model was chosen at 48 epochs when the total loss for the validation set hit the lowest point of 1.6. It was also where the classification accuracy for the validation set hit the highest point of 0.77.

### Deep Learning System Performance to Detect Cancer Cells

**Figure 3:**
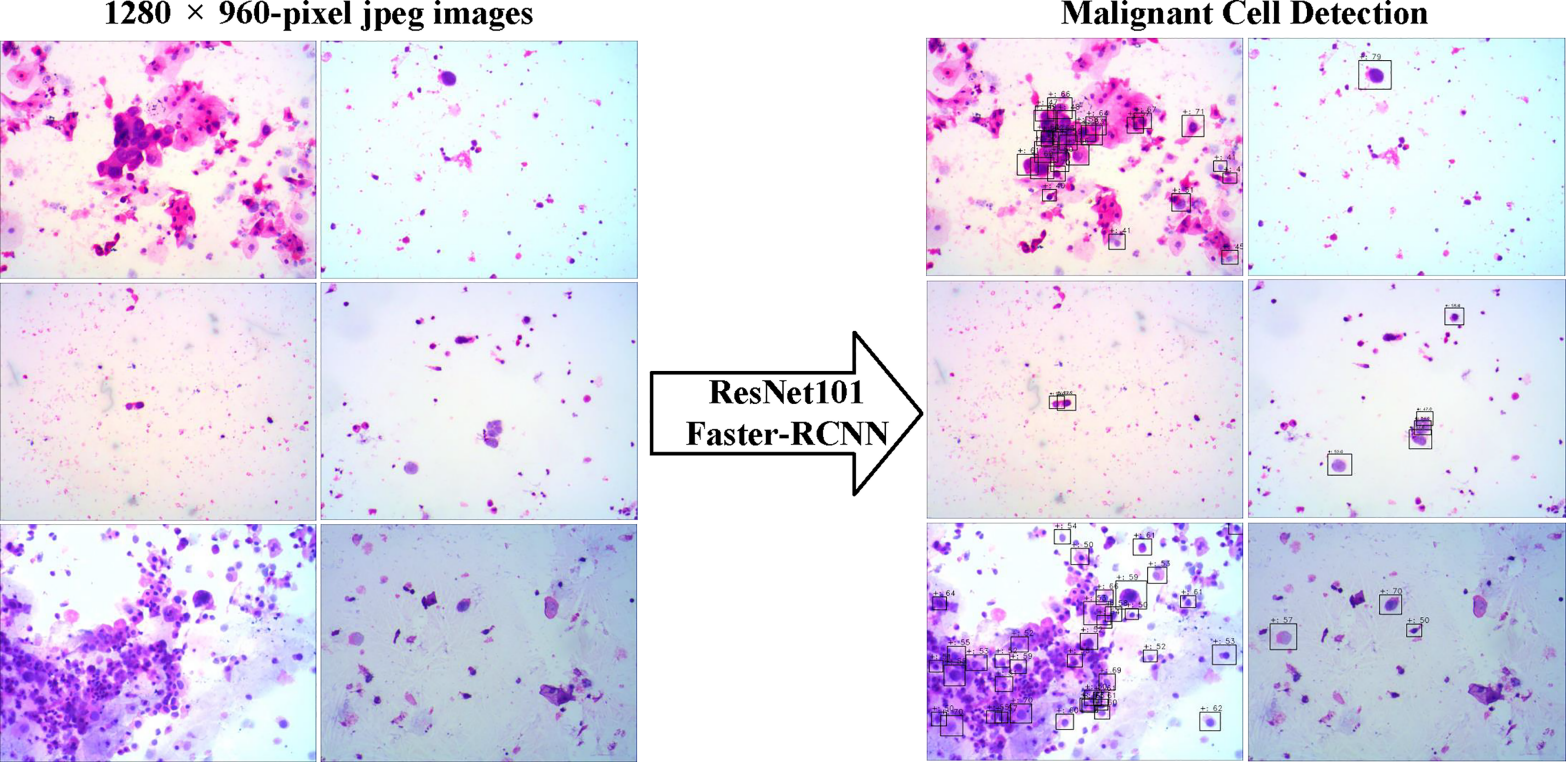
The examples of snapshot images from positive cases and the results by the deep learning system were provided. The malignant cells detected were labeled by the Faster-RCNN, and the possibilities of each detection were also shown.

We evaluated the ability of the DLS to detect cancer cells in the three test sets (**Figure 3**). We took advantage of the value of possibility generated by the DLS and adopted it as the threshold for cell detection.

**Figure 4:**
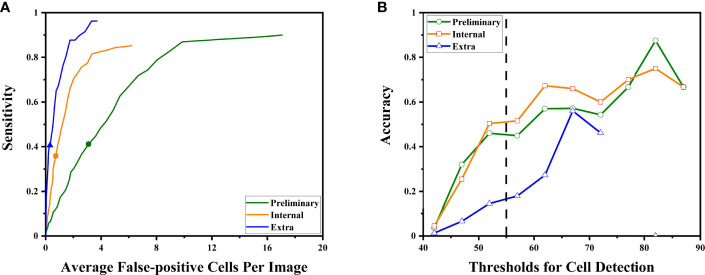
The DLS performance to detect malignant cells is illustrated. **(A)** As the system tried to find more malignant cells (to achieve higher sensitivity), it made more mistakes classifying benign cells as malignant. The sensitivities at the optimal threshold for three sets were marked. **(B)** The accuracy at different thresholds. Thresholds were represented by their middle value (for example, *x* = 52.5 represented the interval of 50–55 points). Higher thresholds tend to have better detection accuracy. The performance at the optimal threshold of 50–55 points is highlighted by the dashed line. At this point, the increasing rates began to slow down.

For all sets, sensitivities increased at the cost of more cells mistakenly spotted as malignant by the DLS (**Figure 4A**). The accuracy initially increased with the thresholds for cell detection, and the rate of increase slowed down by approximately 50–55 (**Figure 4B**). Such a trend was observed in both preliminary and internal tests. Therefore, we chose 55 as the optimal threshold. Under this threshold, the sensitivity is 41% for the preliminary test at the cost of an average of 3.09 false-positive cells per image, 36% for the internal test at the cost of an average of 0.72 false-positive cells per image, and 41% for the extra test at the cost of an average of 0.31 false-positives cell per image (**Figure 4A**). The accuracy of cancer cell detection was 50.0, 50.3, and 14.5 for the preliminary test, internal test, and extra test, respectively (**Figure 4B**).

For the subgroups of the extra test set, results are also calculated under the optimal threshold of 55: for the positive 64 images, sensitivity is 41% with an average of 0.95 false-positive cells per image; for the 281 negative images, the average false-positive cell per image is 0.16 while the sensitivity is not available due to no true positive (cancer cell).

### Deep Learning System Performance to Predict Malignancy

We evaluated whether the exact same DLS can predict malignancy with no further transfer learning. Because the gold standard of malignancy is histopathology, we paired cytology with its corresponding surgical pathology. A total of 97 1,280 × 960-pixel images in the internal test set and 345 1,280 × 960-pixel images in the extra test set that could be paired with corresponding histopathological specimens were used. Here, we proposed a hypothesis that a case with positive cytology was more likely to have malignant surgical pathology. Therefore, the maximal value of possibility (the threshold for cell detection) in each image was adopted as the threshold for malignancy prediction.

**Figure 5:**
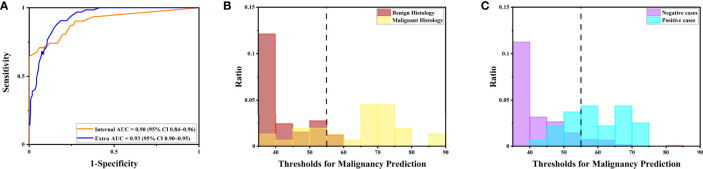
The DLS performance to predict malignancy is illustrated. **(A)** The receiver operating characteristics curve for the performance to predict malignancy for the internal and extra tests. **(B)** The distribution of maximal possibilities in the internal test set. Most images from benign histology were scored below 55. **(C)** The distribution of maximal possibilities in the extra test set. Most images from negative cases were also scored below 55 (highlighted by the dashed line).

Notably, the DLS was able to predict malignancy through cytology images. For the internal test, the AUC was 0.90 (95%CI 0.84–0.96) (**Figure 5A**). The highest kappa score is 0.71 at the threshold of 57 for malignancy prediction, and the highest F1 score is 0.78 at the threshold of 58 (**Table 3**). For the extra test, the AUC was 0.93 (95%CI 0.90–0.95) (**Figure 5A**). The highest kappa score is 0.60 at the threshold of 52 for malignancy prediction, and the highest F1 score is 0.69 at the threshold of 52 (**Table 4**).

**Table 3:** Performance on the internal test set.

Threshold	Sensitivity	Specificity	F1 Score	Kappa Score
40.00	0.94	0.61	0.67	0.45
42.00	0.94	0.62	0.67	0.51
43.00	0.90	0.68	0.70	0.54
44.00	0.90	0.71	0.72	0.56
45.00	0.90	0.73	0.71	0.55
46.00	0.87	0.74	0.72	0.57
47.00	0.87	0.76	0.73	0.57
49.00	0.81	0.79	0.71	0.58
50.00	0.81	0.80	0.69	0.56
51.00	0.74	0.83	0.71	0.56
53.00	0.74	0.88	0.72	0.64
54.00	0.71	0.91	0.75	0.68
55.00	0.71	0.94	0.77	0.68
57.00	0.68	0.95	0.76	0.71
60.00	0.65	1.00	0.78	0.71
65.00	0.61	1.00	0.76	0.68
68.00	0.48	1.00	0.59	0.50
69.00	0.42	1.00	0.56	0.46
70.00	0.39	1.00	0.41	0.32
71.00	0.26	1.00	0.32	0.25
73.00	0.19	1.00	0.32	0.25
75.00	0.16	1.00	0.23	0.17
76.00	0.13	1.00	0.23	0.17
78.00	0.10	1.00	0.18	0.13
83.00	0.06	1.00	0.12	0.09
88.00	0.00	1.00	NA	NA

NA, not available.

**Table 4:** Performance on the extra test set.

Threshold	Sensitivity	Specificity	F1 Score	Kappa Score
40.00	1.00	0.56	0.52	0.35
41.00	1.00	0.59	0.54	0.37
42.00	0.98	0.62	0.57	0.42
43.00	0.98	0.67	0.58	0.44
44.00	0.97	0.69	0.60	0.47
45.00	0.97	0.72	0.62	0.50
46.00	0.94	0.75	0.63	0.51
47.00	0.91	0.78	0.67	0.56
48.00	0.91	0.81	0.68	0.58
49.00	0.88	0.84	0.68	0.59
50.00	0.86	0.85	0.68	0.60
51.00	0.83	0.86	0.68	0.60
52.00	0.80	0.88	0.69	0.60
53.00	0.77	0.89	0.66	0.58
54.00	0.72	0.90	0.67	0.59
55.00	0.67	0.92	0.66	0.58
56.00	0.63	0.94	0.64	0.57
57.00	0.59	0.94	0.61	0.54
58.00	0.55	0.95	0.58	0.51
59.00	0.48	0.96	0.55	0.48
60.00	0.45	0.96	0.53	0.45
61.00	0.42	0.96	0.52	0.45
62.00	0.41	0.96	0.53	0.47
63.00	0.39	0.98	0.50	0.44
64.00	0.36	0.98	0.49	0.44
65.00	0.34	0.99	0.47	0.41
66.00	0.31	0.99	0.37	0.32
67.00	0.23	0.99	0.24	0.20
68.00	0.14	1.00	0.24	0.20
70.00	0.13	1.00	0.19	0.16
71.00	0.11	1.00	0.14	0.11
72.00	0.08	1.00	0.09	0.07
73.00	0.05	1.00	0.06	0.04
74.00	0.03	1.00	NA	NA
78.00	0.00	1.00	NA	NA

NA, not available.

Under the optimal threshold (threshold=55), the DLS also achieved good performance. For the internal test, 4 images that scored higher than 55 have benign histologic results (**Figure 5B**). Thus, sensitivity is 71% (95%CI 52%–85%); specificity is 94% (95%CI 84%–98%); the F1 score is 0.76; and there was a substantial agreement with the reference standard (kappa = 0.68 [95%CI 0.52–0.84]). For the extra test, 22 images that scored higher than 55 have benign histologic results (**Figure 5C**). Thus, sensitivity is 67% (95%CI 54%–78%); specificity is 92% (95%CI 88%–95%); the F1 score is 0.66; and there was a moderate agreement with the reference standard (kappa = 0.58 [95%CI 0.46–0.70]).

## Discussion

In this study, we developed a DLS to predict the likelihood of the presence of tissue malignancy through urine cytopathology. Notably, the system achieved an AUC of 0.90 for the internal test and of 0.93 for the extra test. Under the optimal threshold, sensitivity is 71%, and specificity is 94% for the internal test; sensitivity is 67%, and specificity is 92% for the extra test. These results proved that the DLS was able to predict the presence of malignant tissue merely from urine cytology images.

**Figure 6:**
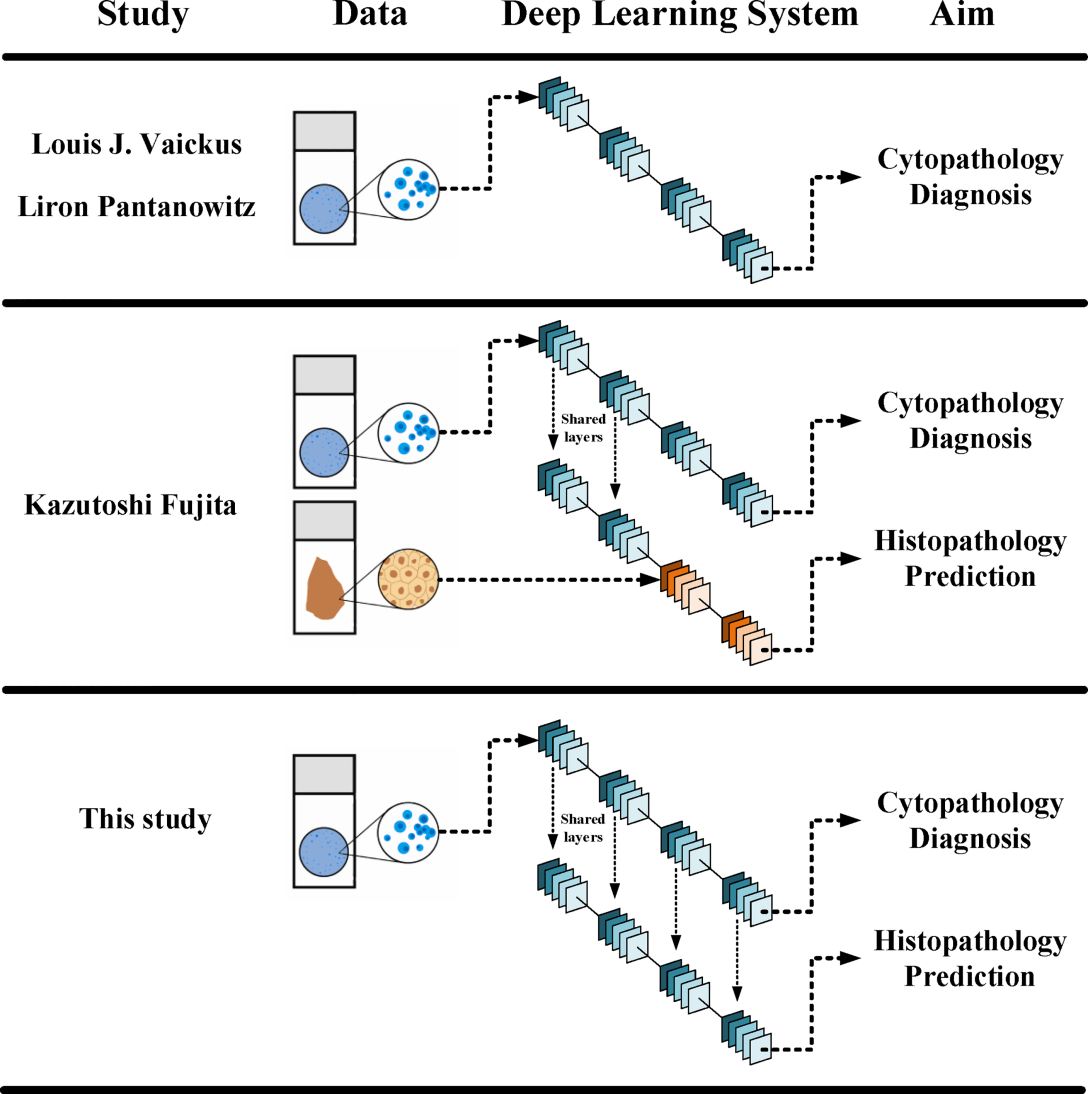
Different designs in between different DLSs. The current DLS could make both cytopathological diagnosis and histopathological prediction, yet it did not need histopathological data in its training.

It has been fully demonstrated that deep learning models can be used to establish a cytology diagnosis system. Vaickus et al. were the first to show that the analysis of urine cytology specimens could be reliably automated. They achieved an accuracy of more than 90% using a hybrid deep-learning and morphometric algorithm (9). Pantanowitz et al. further proved this idea using a much larger data set. They used a pure neural network to exploit and integrate both slide-level and cell-level features and achieved a sensitivity of 79.5% and a specificity of 84.5% for cytopathological diagnosis (10). For both studies, features were carefully engineered to ensure biological interpretability and reproducibility. Features such as the nuclear-cytoplasm ratio, chromatin quality, and the quantity of cells were included (**Figure 6**). Such a design made the system explainable and enables it to fulfill the aim to assist pathologists in cytology reading.

Although the principal indications for the use of cytology include the diagnosis, follow-up, and monitoring of patients with urothelial tumors, the gold standard for the diagnosis of tumor is still histopathology. Therefore, the cyto-histo correlation has become the bottleneck for the use of cytology in clinical settings. Previous studies have shown that deep learning techniques could discern subtle differences in image features that are not readily noticeable to pathologists between tissues from patients with different genetic subtypes, cancer grades, and survival (6–8). Thus, it was reasonable to wonder if deep learning could also distinguish differences in cytology images with different histopathological results.

Fujita et al. were the first to observe that the DLS could not only accurately detect UC cells but also distinguish characteristics traditionally determined using histopathology. They probed two specific characters: whether the lesions were invasive and whether the lesions were high grade. For both, the DLS achieved an AUC higher than 0.86 and F1 score higher than 0.82 (11). The study used a mixing-training model with the first half trained with cytopathological data and the other half trained with histopathological data (**Figure 6**). The results proved that at least part of the features could be shared for cytopathology diagnosis and histopathology prediction. This laid the foundation for a complete cyto-histo correlation.

For the purpose of proving this correlation more stringently, we improved the design of this study on the basis of previous ones. First, we trained the system with only positive cytology images instead of adding negative images as in previous studies. Therefore, this design could insure all the features used by the DLS derived from malignant cases. Second, during malignancy prediction, we did not train the initial DLS again. Instead, we added an additional classifier at the end of the initial DLS. This design rendered two models that shared a same set of convolutional network and detector and, thus, a same set of features. The additional classifier was designed based on the biological meaning of the degree of fit calculated by the DLS. During cancer cell detection, the degree of fit was the likelihood of a cell to be a cancer cell. Therefore, the maximal degree of fit in a certain image represented the likelihood of a case to get malignancy. There were no manually designed features in the DLS. Therefore, the process was explainable since the prediction of malignancy was directly based on the abnormal cells selected by the model and can be verified by examining those candidate abnormal cells in each image. Thus, the design made the DLS not only possible to testify the hypothesis better but also explainable in a unique way.

The test sets in this study included preliminary, internal, and extra test sets. The extra test set came from patients whose cytology was initially diagnosed as negative but later proved to have malignant histopathological results. Overlooked cancer cells in these images were carefully revised in the blinded review at the beginning of this study. Results showed that the extra test set had higher AUC than the internal test set during malignancy prediction. This indicated that the DLS had a good performance for the extra test despite the fact that these cases were among the most difficult to diagnose by traditional cytology. However, the sensitivity and specificity for the extra test under the optimal threshold were lower than that for the internal test. Moreover, during cancer cell detection, the accuracy for the extra test was also lower than that for the preliminary and the internal tests under the optimal threshold, and the accuracy for the extra test was still increasing after the threshold reached the optimal threshold. This indicated that the current optimal threshold using in this study, which was chosen based on the preliminary and internal tests, might not serve as the optimal threshold for the extra test. Future studies are needed for a better strategy to find the optimal threshold for DLS application. At the same time, it is also important to understand that pathologists are not able to spot every true cancer cell since no cytopathological scoring system used for cytology diagnosis at present is perfect. A DLS learnt from pathologists’ annotations inevitably inherited these bias and errors. Therefore, as pathologists had failed to perform excellent in the extra test set themselves, the DLS would only detect cancer cells with additional difficulties.

The results of the DLS performance to predict malignancy showed a relatively high specificity. The sensitivity, however, was not as good as the specificity. This indicated that most cases predicted to have tissue malignancy were indeed patients with UC, while some patients with UC were not successfully identified. This may be attributed to the fact that the DLS still needs further improvement or that some UCs do not present morphologically abnormal cells in urine. Meanwhile, it was not necessary to spot every cancer cell to make a prediction. Instead, it was adequate to find most of the cancer cells while mistaking as few normal cells as possible. These successfully spotted cancer cells were likely to be among the most atypical and, thus, gave the highest scores in an image. Biologically, it was also the patients whose cells in cytology had a higher degree of atypia who were more likely to get UC. Future studies are needed to identify UC in those patients without abnormal cells in urine.

There is currently no gold standard for cyto-histo correlation in urine. Many argue that a negative cytology with a concurrent positive surgical result is not a false negative. Similarly, a positive urine followed by a negative surgical result is not a false positive. However, results in this current study imply that when the DLS predicts a malignant state, it might focus on characteristics that are partially same with those used for UC cell detection. This indicates that there are features on cytology images correlated to histopathology results. Nevertheless, due to the lack of technical maneuver to untangle representative features in Faster-RCNN, we are not able to define each feature and apply them in classical cytology. This is one of the limitations of this study. Another limitation is that this is a retrospective study in a single center. Multicentered prospective studies are warranted to further prove these findings.

Collectively, the current results demonstrated that DLS cytology could be used to predict the likelihood of a case to have histological confirmed malignancy through a cyto-histo correlation. If a DLS can serve as a risk-stratification tool to distinguish clinically relevant malignancy at the time of cytology, urologists can plan in time therapeutic strategies at lower cost that benefit more patients.

## Data Availability Statement

The original contributions presented in the study are included in the article/**Supplementary Material**. Further inquiries can be directed to the corresponding author.

## Ethics Statement

The studies involving human participants were reviewed and approved by the ethics committee of biological and medical research of Peking University First Hospital. Written informed consent for participation was not required for this study in accordance with the national legislation and the institutional requirements.

## Author Contributions

YL, SJ, and WY set up the experimental design. YL, QS, and YF collected and selected the data. SJ was mainly responsible for the high-performance computing and statistical analysis. QS was responsible for annotations and the morphology interpretation in the cytology and histology. LC provided the basic algorithm model. LC and SF provided guidance on deep learning and computer vision techniques. YL wrote the manuscript. WY and HP were responsible for conception, and they supervised the work. All authors reviewed the manuscript and agree with its contents.

## Funding

This study was funded by National Natural Science Foundation of China (81870518 to WY and 62073012 to HP).

## Conflict of Interest

Authors YL, SJ, QS, LC, SF, YF, HP, and WY are on a pending patent (China 202110493539.5). Author LC was employed by the company Yizhun Medical AI Co. Ltd.

## Publisher’s Note

All claims expressed in this article are solely those of the authors and do not necessarily represent those of their affiliated organizations, or those of the publisher, the editors and the reviewers. Any product that may be evaluated in this article, or claim that may be made by its manufacturer, is not guaranteed or endorsed by the publisher.
