# Supplementary material for: A Deep Learning System to Predict the Histopathological Results From Urine Cytopathological Images
Source: Front Oncol. 2022 May 24;12:901586. doi: 10.3389/fonc.2022.901586 (PMC9170952; doi:10.3389/fonc.2022.901586)

We randomly cropped 175×200 pixel sub-images containing at least one pathologist's annotation from the original images. The selected sub-images were randomly split into a training set and a validation set by a ratio of 5:1. Spatial augmentation, including 90° rotation and vertical and horizontal flip, was implemented.

To achieve better performance, we adopted the state-of-the-art two-stage-network

Faster R-CNN to detect malignant cells. By unifying the detection process and

sharing computation across all regions of interest, Faster R-CNN allows end-to-end

training to generate high-quality results. The Faster R-CNN model consists of three

parts (Figure A): a feature extraction network to generate feature maps from the

original image, a region proposal network to extract regions from the feature maps,

and a classifier to classify the candidate object. We implemented ResNet101 as

the feature selection network, using the parameters pre-trained on ImageNet to generate feature maps. The classifier categorised each candidate object into

one of the four following categories: "+", "+-", "@", and "bg" ("background"). However only the “+” cells were visualized on the outputs.

The loss function of the model consisted of four components. The classification

loss and the bounding box regression loss from the region proposal network gave an

initial prediction of each potential object. The classifier output the final decision along

with the refined bounding box by repeatedly applying the same two losses on each

generated proposal. In our dataset, the samples of "+" class cells were around two

times the "+-" class. To mitigate the effect of this imbalance, we used focal loss for

classification. An Adam optimiser was used for optimisation with a learning rate of

0.0001. After each epoch, the performance of the model for the validation set was

measured to assess the training of the system. Training was stopped if there was no

improvement after several rounds. Both the total loss and the system accuracy stabilised after 45–50 epochs for the validation set. A final model was chosen when the total loss for the validation set hit the lowest point of 1.6 at 48 epochs. It was also where the classification accuracy for the validation set hit the highest point of 0.77 (Figure B).

The model was implemented in Python 3.8 using TensorFlow (1.12.0) and Keras

(2.0.3). For more details, please see our GitHub page at https://github.com/moyiliyi/keras-faster-rcnn.

Many examples of true and false positive cells detected by the system were shown here. We stacked them together while maintained their relative sizes on original images (Figure C).

A


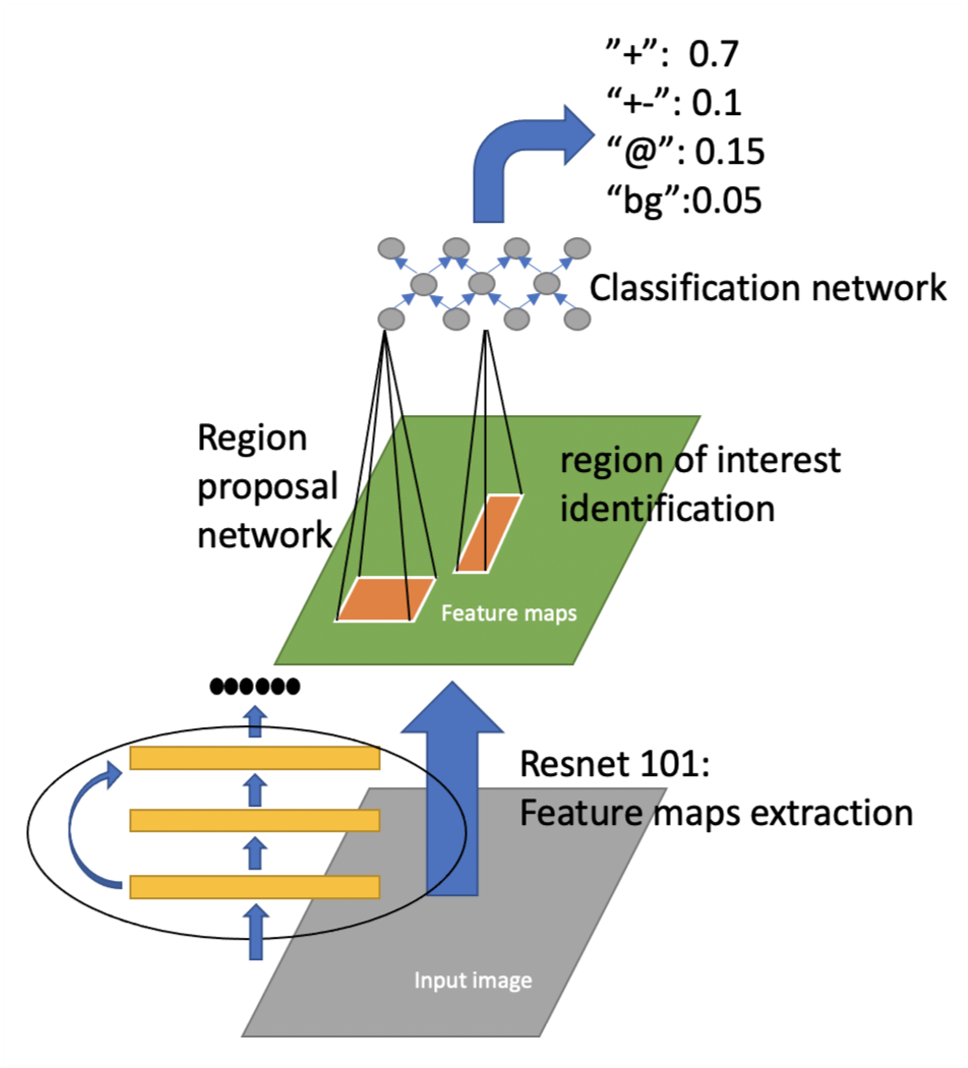


B


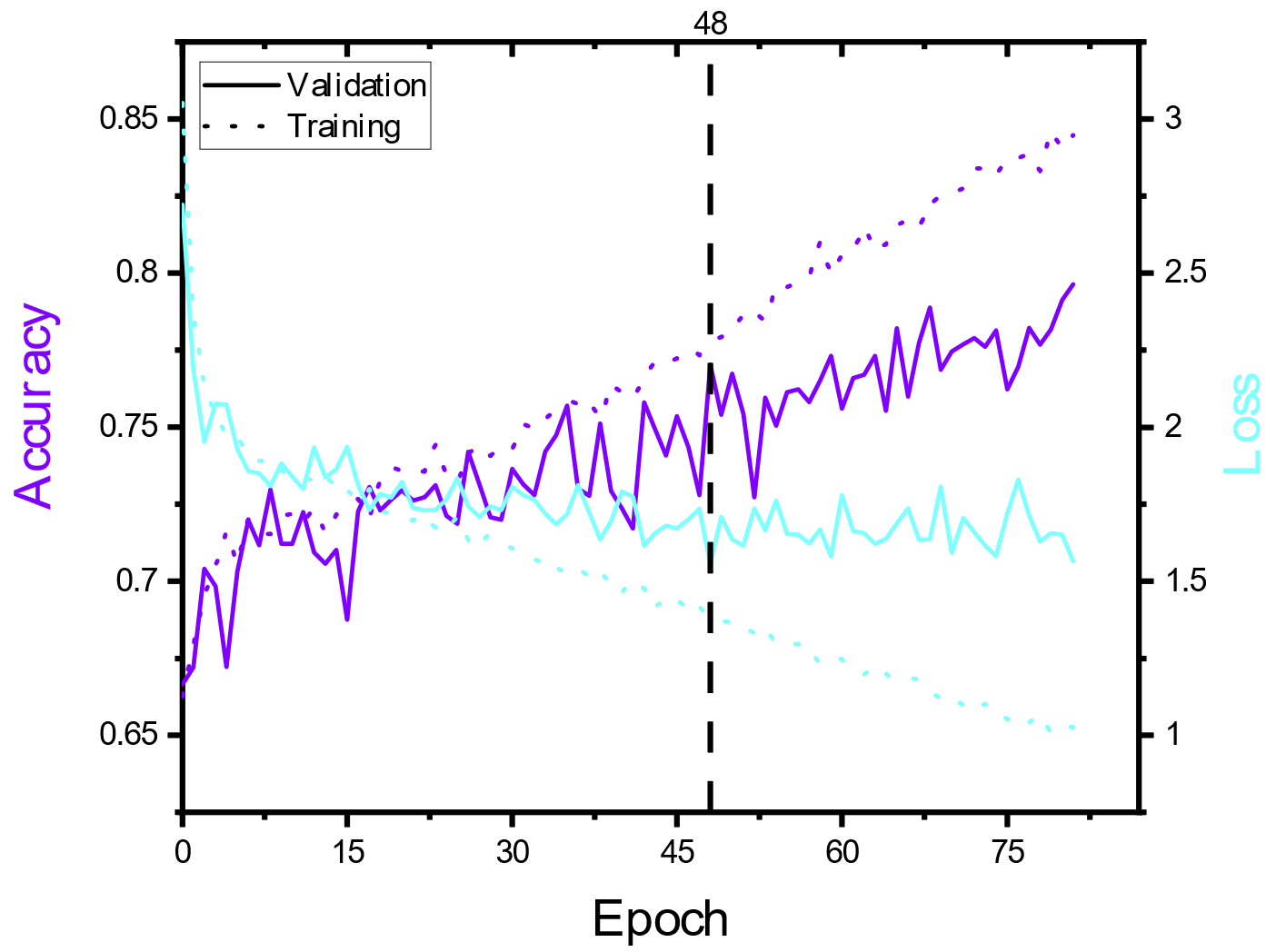


C


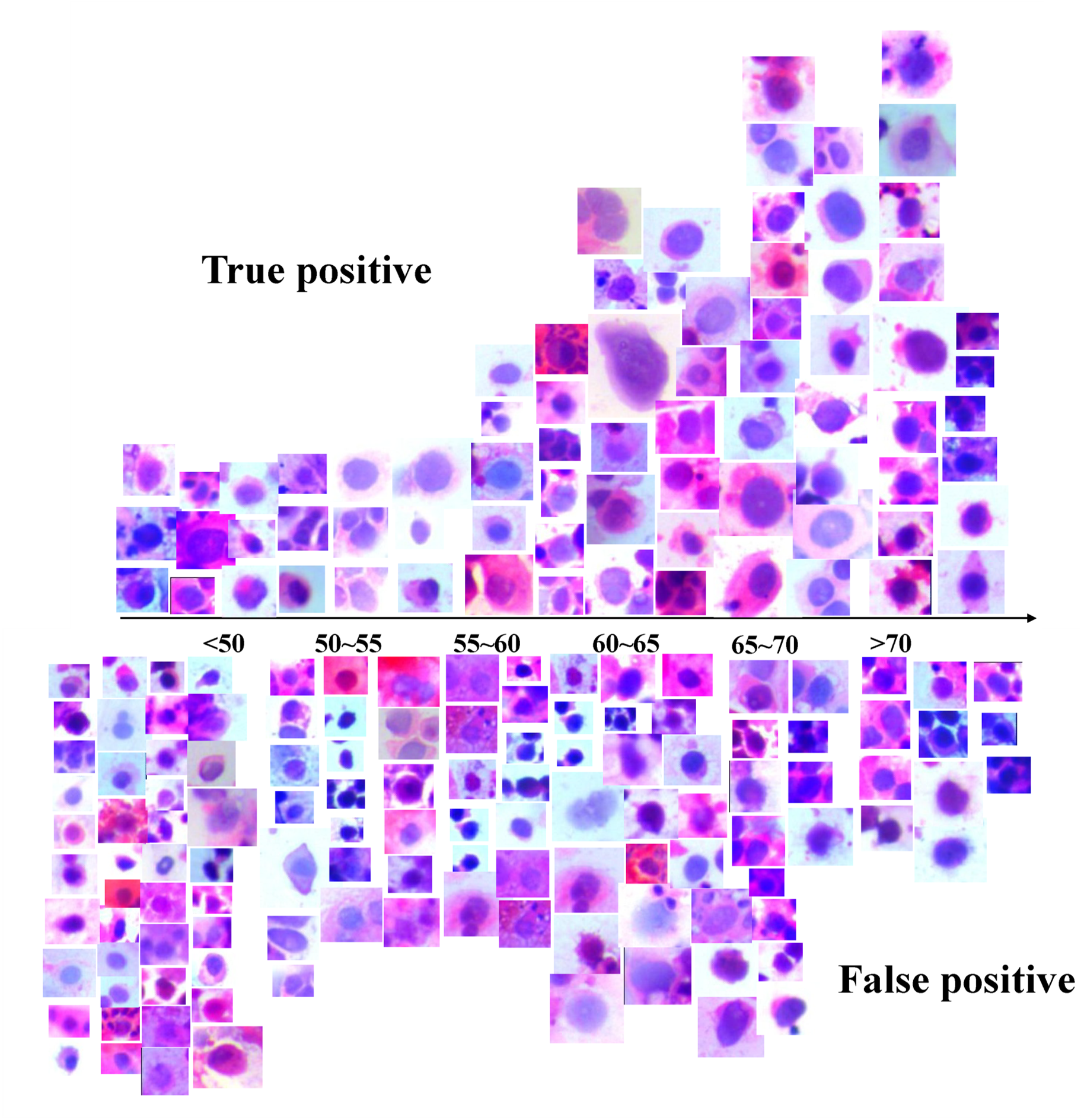

Supplement: Supplementary file 1 [file DataSheet_1.docx]
